# Elacestrant in metastatic breast cancer: Is the “standard of care” meeting standard requirements?

**DOI:** 10.1016/j.tranon.2021.101273

**Published:** 2021-11-16

**Authors:** Timothée Olivier, Vinay Prasad

**Affiliations:** a Department of Oncology, Geneva University Hospital, 4 Gabrielle-Perret-Gentil Street, Geneva 1205, Switzerland; b Department of Epidemiology and Biostatistics, University of California San Francisco, 550 16th St, 2nd Fl, San Francisco, CA 94158, USA

**Keywords:** Hormone sensitive breast cancer, Substandard control arm, Hormonotherapy, Metastatic

## Abstract

The EMERALD trial was an open label phase 3 trial evaluating elacestrant, the first oral selective estrogen receptor degrader (SERD), as compared to “standard of care”, in ER+/HER2- (hormone receptor positive, no HER2 overexpression) advanced or metastatic breast cancer.

The EMERALD trial restricted the “standard of care” control arm to limited options that may have led to a substandard control arm. We describe how the EMERALD trial protocol allowed different clinically inappropriate scenarios in the control arm, according to prior therapy. The main relevant question remains the potential advantage of elacestrant over fulvestrant in fulvestrant-naive patients.

Analyzing outcomes in subgroups according to prior and per-protocol therapy would help analyzing trial results. However, these subgroup results may be non-significant, and another randomized trial will be needed. Trials should be designed to answer directly clinical questions that are relevant.

## Highlights

• In randomized controlled trial, the “standard of care” should not be restricted as it may penalize the control arm.
• Restricting the control arm can lead to clinically inappropriate situations according to prior and per-protocol treatment.
• Trial designs should allow us to answer clinical questions that are directly relevant to real-life practice.

## Graphical abstract

**Figure:**
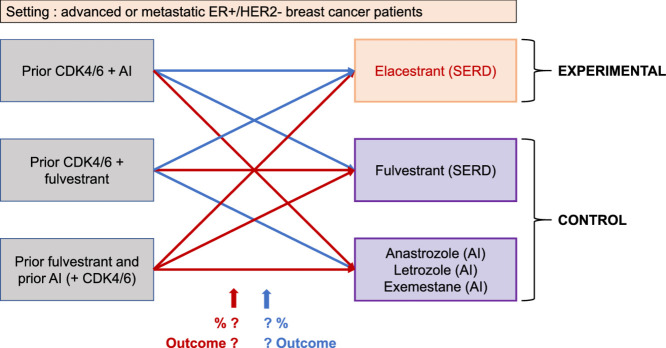

On October 20th, 2021, Menarini Group and Radius Health announced positive phase 3 results from the EMERALD trial evaluating elacestrant in ER+/HER2- (hormone receptor positive, no HER2 overexpression) advanced or metastatic breast cancer [1]. The EMERALD trial (NCT03778931), is an open label phase 3 trial, investigating elacestrant, the first oral selective estrogen receptor degrader (SERD), against “standard of care”, in advanced or metastatic ER+/HER2- breast cancer patients [2].

To be enrolled, patients must have received one or two lines of endocrine therapy for advanced or metastatic breast cancer, and have received prior treatment with a CDK4/6 inhibitor in combination with either fulvestrant or an aromatase inhibitor. Patients could have received no more than one line of chemotherapy (in the advanced or metastatic setting). Primary endpoints were progression free survival (PFS) in the estrogen receptor, ESR-1 mutated patients and PFS in all patients (intention to treat population). ESR-1 mutations is described as a resistance mechanism occurring under endocrine therapy such as tamoxifen or aromatase inhibitors [3].

A press release has touted that EMERALD trial met both primary endpoints, showing statistical improvement in PFS in the intention to treat population as well as in the ESR-1 mutated group of patients. Submissions for 2022 regulatory approvals by the FDA (US) and the EMA (Europe) are ongoing [1]. Although we are excited about the option of a first in class, oral selective estrogen receptor degrader, open questions remain regarding the design and interpretation of this study.

The control arm of the EMERALD trial is referred as “standard of care”. The expression “standard of care” is applied generously, as the control arm is restricted to only four options: fulvestrant, anastrozole, letrozole, exemestane. Among these options, one is a selective estrogen receptor degrader (SERD), being fulvestrant, the three others are aromatase inhibitors (AI). Elacestrant is the first oral selective estrogen receptor degrader (SERD). The first in class SERD is fulvestrant, that is given via intramuscular route, and was approved in 2002 in post-menauposal women with disease progression following antiestrogen therapy.

We identified several situations, allowed by the EMERALD trial protocol, in which the “standard of care” would actually lead to a substandard control arm (Fig. 1).

(1) A patient that received fulvestrant with a CDK4/6 inhibitor at first- or second-line treatment should not receive fulvestrant at progression.
(2) A patient that received an AI with a CDK4/6 inhibitor as a first- or second- line treatment should never be proposed an AI monotherapy at progression.
(3) A patient that progressed after AI and fulvestrant should not receive either the same treatment on which the progression occurred, so the control arm is not a valid option.
(4) Lastly and logically, the same patients as in point (3), presenting progression after AI and fulvestrant, because they have no valid option in the control arm, should not either be randomized to the experimental arm; they should be excluded from the trial.

**Fig. 1:**
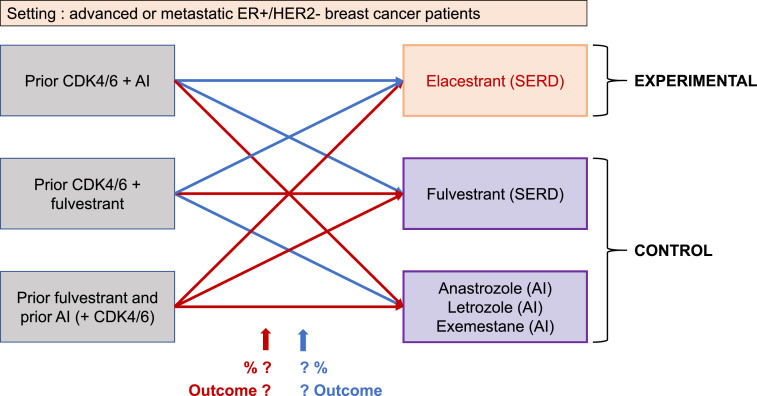
Pre-protocol and per-protocol paired clinical situtations represented by arrows: blue arrows = acceptable, red arrows = inappropriate (For interpretation of the references to color in this figure legend, the reader is referred to the web version of this article.).

All these situations were theoretically allowed by the protocol.

The press-released announced: “A full evaluation of the data is ongoing. Current plans are to have those results presented at the upcoming San Antonio Breast Cancer Symposium in December 2021 and to publish them in a peer-reviewed journal.” We hope the data that really matters will be available: did elacestrant was better than fulvestrant in fulvestrant-naive patients? However, it is possible that these subgroup results are non-significant and a separate randomized trial will need to be run for this question.

No potential conflict of interest relevant to this letter was reported.

## CRediT authorship contribution statement

**Timothée Olivier:** Conceptualization, Writing – original draft, Writing – review & editing. **Vinay Prasad:** Conceptualization, Writing – review & editing.

## CRediT authorship contribution statement

**Timothée Olivier:** Conceptualization, Writing – original draft, Writing – review & editing. **Vinay Prasad:** Conceptualization, Writing – review & editing.

## Declaration of Competing Interest

Vinay Prasad's Disclosures: Research funding: Arnold Ventures; Royalties: Johns Hopkins Press, Medscape; Honoraria: Grand Rounds/lectures from universities, medical centers, non-profits, and professional societies; Consulting: UnitedHealthcare; Speaking fees: Evicore; Other: Plenary Session podcast has Patreon backers. Timothée Olivier have no financial nor non-financial conflicts of interest to report.
